# A rare case of Epstein–Barr virus-positive mucocutaneous ulcer that developed into an intestinal obstruction: a case report

**DOI:** 10.1186/s12876-020-1162-2

**Published:** 2020-01-13

**Authors:** Nozomi Morita, Chiaki Okuse, Keigo Suetani, Hiroyasu Nakano, Tetsuya Hiraishi, Shinya Ishigooka, Shuzo Mori, Tsukasa Shimamura, Takeshi Asakura, Junki Koike, Fumio Itoh, Michihiro Suzuki

**Affiliations:** 1 Division of Gastroenterology and Hepatology, Department of Internal Medicine, Kawasaki Municipal Tama Hospital, 1-30-37, Shukugawara, Tama-Ku, Kawasaki, Japan; 2 Division of Gastroenterological and General Surgery, Department of surgery, Kawasaki Municipal Tama Hospital, 1-30-37, Shukugawara, Tama-Ku, Kawasaki, Japan; 3 Department of Pathology, Kawasaki Municipal Tama Hospital, 1-30-37, Shukugawara, Tama-Ku, Kawasaki, Japan; 4 Division of Gastroenterology and Hepatology, Department of Internal Medicine, St. Marianna University School of Medicine, 2-16-1 Sugao, Miyamae-Ku, Kawasaki, Japan

**Keywords:** Epstein–Barr virus-positive mucocutaneous ulcer (EBV-MCU), Immunosuppression, Aging, Intestinal obstruction, Surgical resection

## Abstract

**Background:**

Epstein–Barr virus-positive mucocutaneous ulcer (EBV-MCU) is a new category of mature B-cell neoplasms. Ulcers occur in the oropharyngeal mucosa, skin, and gastrointestinal tract. The onset of EBV-MCU is suggested to be related to the decreased immunity of the patient, the causes of which include the use of immunosuppressive agents and aging. EBV-MCU may regress spontaneously and it often has a benign course after the dose reduction or discontinuation of immunosuppressive agents or during follow-up. Here, we report the case of a patient who required surgical resection for the intestinal obstruction arising from EBV-MCU.

**Case presentation:**

A Japanese elderly male visited our hospital with chief complaints of a palpable mass and dull pain in the left upper quadrant, loss of appetite, and weight loss. Although abdominal computed tomography and total colonoscopy (TCS) revealed a tumor with circumferential ulcer in the transverse colon, histopathological analysis of a biopsy specimen of this lesion showed only nonspecific inflammation. Because the tumor spontaneously regressed during the time he underwent tests to obtain a second opinion from another hospital, TCS was reperformed on the patient. TCS revealed that the tumor decreased in size and the inflammatory changes in the surrounding mucosa tended to improve; however, tightening of the surrounding mucosa due to scarring was observed. Another histopathological analysis of a biopsy specimen showed widespread erosion of the mucosa and the formation of granulation tissue with marked infiltration of various inflammatory cells into the mucosal tissue of the large intestine. Moreover, some of the B-lymphocyte antigen CD20-positive B cells were also positive for EBV-encoded small RNA-1, suggesting the possibility of EBV-MCU. Later, the tumor developed into an intestinal obstruction; thus, the transverse colon was resected. Histopathological analysis of the resected specimen demonstrated scattered Hodgkin and Reed–Sternberg-like multinucleated large B cells in addition to EBER-1-positive cells. The patient was finally diagnosed as having EBV-MCU.

**Conclusions:**

This is the first report of a case of EBV-MCU that developed into an intestinal obstruction requiring surgical resection. It is necessary to consider the possibility of EBV-MCU when examining an ulcerative or tumorous lesion in the gastrointestinal tract.

## Background

Epstein–Barr virus-positive mucocutaneous ulcer (EBV-MCU) is a new category of mature B-cell neoplasms and was formally adopted in the World Health Organization (WHO) classification of lymphoid neoplasms updated in 2016 [1]. The results of previous studies suggest that the onset of EBV-MCU is related to the decreased immunity of the patient, the causes of which include primary or acquired immunosuppression, iatrogenic immunosuppression by immunosuppressive agents, and aging [2–13]. Ulcers occur in the oropharyngeal mucosa, skin, and gastrointestinal tract. They may regress spontaneously and often have a benign course after the dose reduction or discontinuation of immunosuppressive agents or during follow-up [2, 4, 5, 9, 10, 13]. We report the case of an elderly male patient who required surgical resection for the intestinal obstruction arising from EBV-MCU.

## Case presentation

An 81-year-old Japanese male visited our hospital with chief complaints of a palpable mass and dull pain in the left upper quadrant, loss of appetite, and weight loss of 5 kg within two months. He started noticing the mass in the left upper quadrant and the other symptoms in late July 2017. He was admitted to our hospital for detailed examinations and treatment in August 2017.

He had high blood pressure and chronic kidney disease. He had a clear sensorium. His body temperature was 36.7 °C. He showed no yellowing of the bulbar conjunctiva, palpebral conjunctival pallor, or abnormal findings in the skin and intraoral mucosa. There was a palpable elastic, soft mass in the left upper quadrant. Although he felt a dull pain in that area, there was no obvious tenderness. There were no palpable superficial lymph nodes nor abnormalities in the extremities. His laboratory findings on admission are shown in Table 1.

**Table 1:** Laboratory findings on admission

Hematology	
WBC	7400/μl
Stab	8.0%
Seg	62.5%
Lymph	10.5%
Mono	17.5%
Baso	1.5%
RBC	335 × 10^4^/μl
Hgb	11.1 g/dl
Hct	33.3%
Plt	19.3 × 10^4^/μl
Coagulation	
PT	130% (PT-INR 0.85)
APTT	27.1 s
Fibrinogen	606 mg/dl
Biochemistry	
TP	7.3 g/dl
ALB	3.5 g/dl
T.Bil	0.6 mg/dl
AST	21 U/l
ALT	9 U/l
LDH	237 U/l
γ-GTP	25 U/l
ALP	288 U/l
Cr	1.94 mg/dl
BUN	41.6 mg/dl
Na	136 mEq/l
K	4.4 mEq/l
Cl	100 mEq/l
FPG	92 mg/dl
Hgb A1c	5.4%
Serology and others	
CRP	8.39 mg/dl
IgG	1569 mg/dl
IgA	321 mg/dl
IgM	31 mg/dl
Tumor markers	
CA19–9	5.3 U/ml
CEA	1. 8 ng/ml
DUPAN-2	< 25 U/ml
SPAN-1	5 U/ml
IL-2R	2420 U/ml

Abbreviations: *CA19–9* carcinoantigen 19–9; *CEA* carcinoembryonic antigen; *IgA* immunoglobulin A; *IgG* immunoglobulin G; *IgM* Immunoglobulin M; *IL-2R* interleukin-2 receptor

**Fig. 1:**
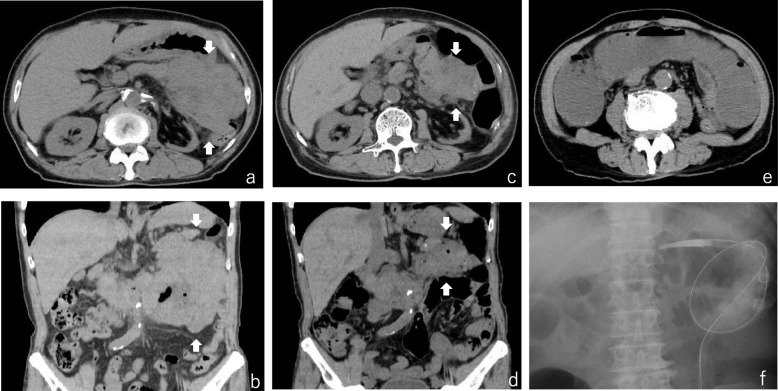
Abdominal computed tomography imaging. A plain abdominal computed tomography (CT) image on admission shows an irregular tumor in the left upper quadrant corresponding to the transverse colon. The tumor was in close contact with the pancreatic body and tail, and the gastric corpus greater curvature. Mildly enlarged lymph nodes were observed at the periphery of the tumorous lesion (1**a** and 1**b**). The second plain abdominal CT conducted 36 days after the first CT showed that the tumor had regressed spontaneously and the swelling of lymph nodes around the tumorous lesion had disappeared (1**c** and 1**d**). CT conducted 48 days after the first visit revealed intestinal distention on the oral side of the narrowed area in the transverse colon, and the patient underwent decompression by transanal ileus tube insertion (1**e** and 1**f**)

Because the results of biochemical tests indicated renal damage, plain abdominal computed tomography (CT) was performed. An irregular tumor of approximately 130 mm × 120 mm × 80 mm in size was observed in the left upper quadrant, which corresponds to the transverse colon. No intestinal distention was observed on the oral side of the tumor. Moreover, the tumor was in close contact with the pancreatic body and tail, and the gastric corpus greater curvature, suggesting invasion into nearby organs. Also, mildly enlarged lymph nodes were observed at the periphery of the tumor (Fig. 1a and b).

**Fig. 2:**
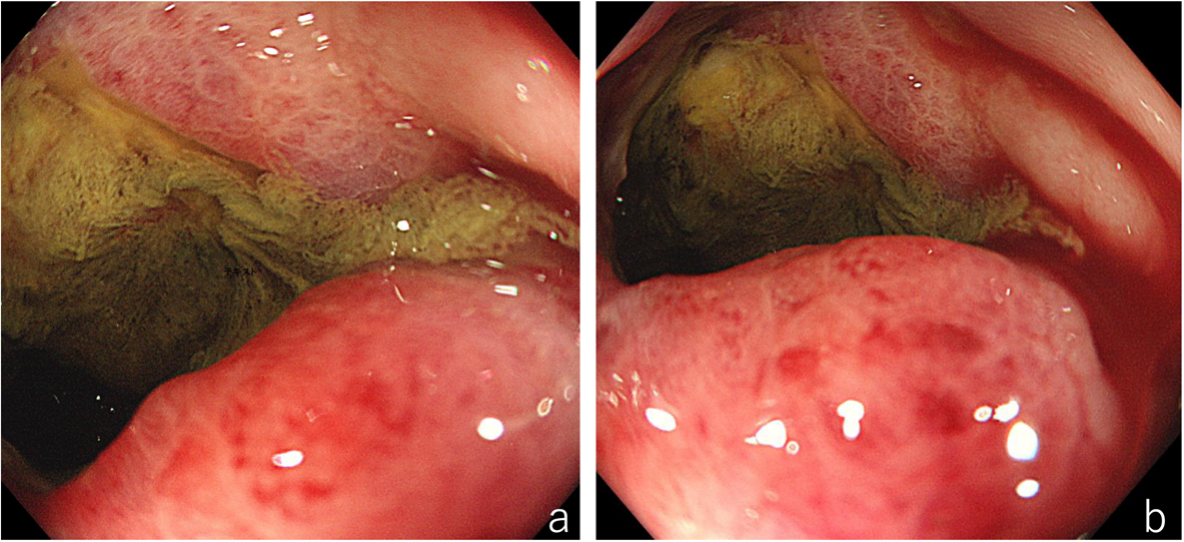
First total colonoscopy. Total colonoscopy (TCS) revealed a circumferential ulcer of the tumor covered with a thick slough in the splenic flexure of the transverse colon. The surrounding mucosa showed marked inflammatory changes and thickening (2**a** and 2**b**)

**Fig. 3:**
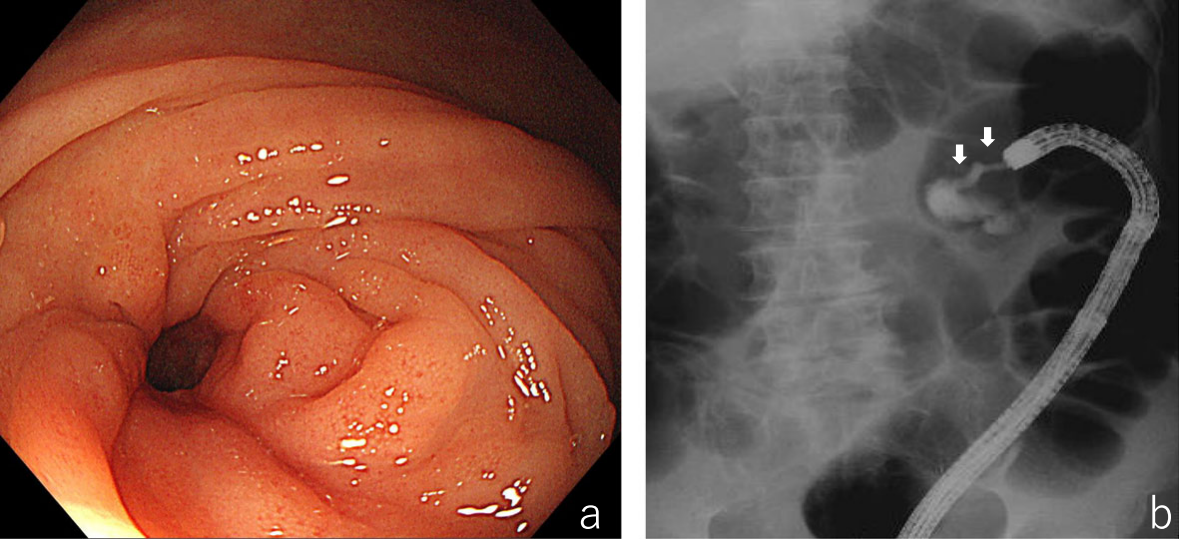
Second total colonoscopy. In the second TCS, the circumferential ulcer of the tumor had decreased in size and the inflammatory changes in the surrounding mucosa trended to improve; however, tightening of the surrounding mucosa due to scarring was observed. There was a continuous narrowing of the lumen over a length of approximately 30 mm (3**a** and 3**b**)

**Fig. 4:**
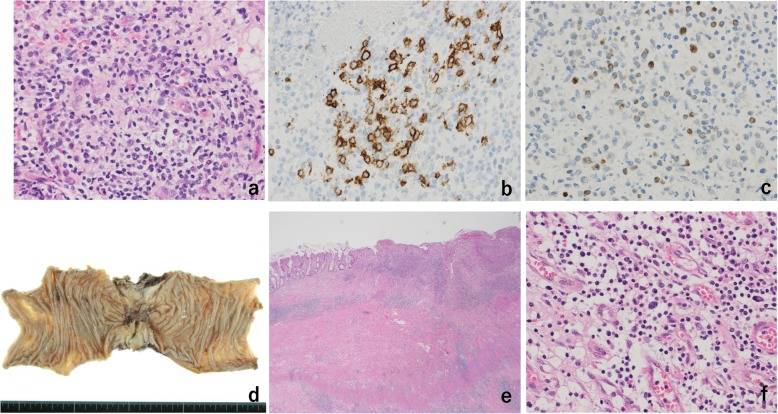
Histopathological analysis. Histopathological analysis revealed widespread erosion of the mucosa and the formation of granulation tissue with marked infiltration of inflammatory cells consisting of small lymphocytes, plasma cells, histiocytes, and medium-size atypical nucleated cells in the mucosal tissue of the large intestine (hematoxylin–eosin stain, original magnification × 40) (**a**). Some of the CD20-positive B cells identified by immunohistochemical analysis (original magnification × 40) (**b**) were also found to be positive for EBER-1 by ISH (original magnification × 40) (**c**). The resected specimen showed an ulcerative lesion with narrowing of the lumen (**d**). The histopathological analysis of the resected specimen revealed marked infiltration of inflammatory cells, consisting mainly of small lymphocytes and plasma cells, as well as fibrosis in all layers of the colon wall (hematoxylin–eosin stain, original magnification × 2) (**e**). HRS-like multinucleated large B cells were also occasionally present (hematoxylin–eosin stain, original magnification × 40) (**f**)

The total *co*lonoscopy (TCS) performed six days after his first visit revealed a tumor with a circumferential ulcer covered with a thick slough in the splenic flexure of the transverse colon. Moreover, the marked thickening and inflammatory changes of the surrounding mucosa made it difficult for the endoscope to pass through, although the lesion had not yet developed into an intestinal obstruction (Fig. 2a and b). Histopathological analysis of several specimens of the tumor was performed, but the findings at that time were only erosion of the mucosa with moderate inflammatory cell infiltration and regenerative changes in the mucosal epithelium. Although a definitive diagnosis was not yet made at that time, we recommended an exploratory laparotomy because of intestinal stenosis. However, he requested a second opinion. While undergoing tests to obtain a second opinion from another hospital, he became aware that the palpable mass decreased in size. Furthermore, a follow-up CT performed after he obtained a second opinion (36 days after the first CT) showed that the tumor had regressed spontaneously and the swelling of small lymph nodes around the tumor had disappeared (Fig. 1c and d). Therefore, TCS was reperformed 41 days after his first visit to our hospital. The tumor had decreased in size and the inflammatory changes in the surrounding mucosa tended to improve; however, tightening of the surrounding mucosa due to scarring (Fig. 3a) and the continuous narrowing of the intestine over a length of approximately 30 mm were observed (Fig. 3b). Histopathological analysis of a biopsy specimen of the periphery of the tumor showed widespread erosion of the mucosa and the formation of granulation tissue with marked infiltration of inflammatory cells consisting of small lymphocytes, plasma cells, histiocytes, and medium-size atypical nucleated cells in the mucosal tissue. Some of the B-lymphocyte antigen CD20-positive B cells (CD20-positive B cells) identified by immunohistochemical analysis were also found to be positive for Epstein–Barr virus (EBV)-encoded small RNA (EBER)-1 by in situ hybridization (ISH), suggesting the high possibility of EBV − positive mucocutaneous ulcer (EBV-MCU) (Fig. 4a, b, and c). Forty-eight days after his first visit, while he was being prepared exploratory laparotomy, he started to suffer from constipation and intermittent abdominal pain. Because plain abdominal CT revealed intestinal distention on the oral side of the narrowed area in the transverse colon, the patient was diagnosed as having intestinal obstruction and underwent decompression by transanal ileus tube insertion (Fig. 1e and f). Therefore, although the definitive diagnosis was not yet confirmed histopathologically, transverse colon segmental resection was performed with the patient’s fully informed consent. The resected specimen showed an ulcer of 35 mm × 25 mm in size with narrowing of the intestine (Fig. 4d). The histopathological analysis of the specimen revealed marked inflammatory cell infiltration and fibrosis in all layers of the colon wall. Most of the infiltrating cells were lymphocytes and plasma cells. Also, Hodgkin and Reed–Sternberg (HRS)-like multinucleated large B cells were found scattered (Fig. 4e and f). A large proportion of CD20-positive B cells identified by immunohistochemical analysis were also found to be positive for EBER-1 by ISH, similarly to the postoperative immunohistochemistry test. No apparent clonal growth of lymphocytes was shown by the flow cytometry of the resected specimen. No dividing cells were identified by G-banding differential staining. Taking these findings together, he was finally diagnosed as having EBV-MCU. No recurrence was observed 20 months after the surgery.

## Discussion and conclusions

In 2010, Dojcinov et al. reported on EBV-positive circumscribed ulcerative lesions occurring in the oropharyngeal mucosa, skin, and gastrointestinal tract of 26 patients with iatrogenic or aging-induced immunosuppression, and they identified these lesions as EBV-MCU [2]. Further study revealed that the clinical features of EBV-MCU are different from that of EBV-positive diffuse large B-cell lymphoma (DLBCL), because of its self-limited growth potential and favorable response to conservative management. Thus, EBV-MCU was formally adopted as a new category independent of the subtypes of DLBCL in the WHO classification of lymphoid neoplasms updated in 2016 [1].

Because no diagnostic criteria have been established yet for EBV-MCU, it is diagnosed on the basis of clinical background factors and histopathological findings [2–13]. It has been reported that the characteristic histopathological findings of EBV-MCU include infiltration of various cells, various immunohistochemical patterns such as CD20 and CD30 positivity, the detection of EBER-positive cells by ISH, among which HRS-like B cells are found [2–13]. In the present case, the initial histopathological findings of the biopsy specimen collected from the lesion were nonspecific, which made the diagnosis difficult. However, repeated histopathological analyses of endoscopically obtained biopsy specimens and that of the resected specimen showed findings that are characteristic of EBV-MCU. It is necessary to consider the possibility of EBV-MCU when performing detailed examinations of an ulcerative or tumorous lesion in the gastrointestinal tract. Also, as in this case, a single histopathological analysis or an analysis with a small biopsy section may not lead to a definitive diagnosis. Thus, it is necessary to perform repeated analyses or to examine a large section.

It is suggested that the onset of EBV-MCU is related to the decreased immunity of a patient, the causes of which include a history of organ transplantation, use of immunosuppressive agents, and aging [2–13]. Roberts et al. reviewed the cases of 51 patients with EBV-MCU (including those of the 26 patients reported by Dojcinov et al.) and reported that the causes of EBV-MCU were the use of immunosuppressive agents (methotrexate, azathioprine, cyclosporine, mycophenolate, or tacrolimus) in 56% of the patients, aging (median age, 80 years; range, 64–101 years) in 40%, and congenital immunosuppression (hypogammaglobulinemia and T-cell deficiency) in 4% [9]. Also, because elderly people accounted for a large proportion of patients who used immunosuppressive agents, they assumed that the main onset factor for EBV-MCU is immunosenescence [9]. In the present case, our patient was 81 years old and had chronic kidney disease as a comorbidity. Advanced chronic kidney disease leads to the development of virus-associated cancer and a decreased vaccination response due to decreased immunity [14]. Immunosenescence, as well as decreased immunity due to aging and chronic kidney disease, is suspected to be the onset factors for EBV-MCU in our patient.

It has been reported that EBV-MCU most commonly occurs in the oropharyngeal mucosa at a rate of 40%, followed by the gastrointestinal tract and skin [9]. As far as we searched, there have been case reports of 13 patients with gastrointestinal tract EBV-MCU to date [2–7, 11, 12]. Table 2 shows a summary of the cases of gastrointestinal tract EBV-MCU, including the present case. There were seven males and seven females whose median age was 64.0 years (range, 26–81 years); note that some of them were young. The lesion locations were the esophagus in three patients, the terminal ileum in one patient, the cecum in one patient, the colon in four patients, the colon and rectum in one patient, and the rectum and anus in four patients. Gastrointestinal tract EBV-MCU tends to occur in the lower gastrointestinal tract. Twelve of the 14 patients (85.7%) had clinical background factors for EBV-MCU, such as collagen disease, inflammatory bowel disease, primary immunosuppression, and a history of organ transplantation. Eleven patients had received immunosuppressive agents. In addition to aging, the intervention with immunosuppressive agents may be largely involved in the pathogenesis of gastrointestinal tract EBV-MCU.

**Table 2:** Summary of reported cases of EBV-MCU associated with gastrointestinal tract involvement

Case	Age	Sex	Lesion location	Comorbidity	Risk factor	Treatment	Outcome	Ref
1	69	F	Colon	RA	MTX	ND	ND	2
2	53	F	Colon, Rectum	CD	MTX, Infliximab	Dose reduction	Development of HL	7
3	75	F	Esophagus	RA	AZA	Dose reduction	Improvement	2
4	63	M	Anus	CD	AZA	Dose reduction	Improvement	5
5	81	F	Colon	ITP	AZA	Surgical resection	Death by LPD	3
6	26	M	Rectum	CD	AZA, Infliximab	Surgical resection	Improvement	12
7	78	M	Rectum	UC	CyA	Dose reduction	Improvement	2
8	64	F	Colon	HSCT	CyA	Dose reduction	Improvement	2
9	61	M	Esophagus	Organ transplant	MMF	Dose reduction	Improvement	4
10	70	M	Rectum	Organ transplant	MMF	Dose reduction, Rituximab adm	Improvement	4
11	32	M	Terminal ileum	Organ transplant	MMF, Tac	Dose reduction, Rituximab adm	Improvement	4
12	61	F	Esophagus	PID	immune deficiency	Rituximab adm, IVIG adm	No change	6
13	64	F	Cecum	None	Age	Surgical resection	Improvement	11
14	81	M	Colon	CKD	Age	Surgical resection	Improvement	Our case

Abbreviations; *adm* administration; *AZA* azathioprine; *CD* crohn’s disease; *CKD* chronic kidney disease; *CyA* cyclosporine; *EBV-MCU* Epstein Barr virus-positive mucocutaneous ulcer; *F* female; *HL* Hodgkin’s lymphoma; *HSCT* hematopoietic stem cell transplantation; *ITP* idiopathic thrombocytopenic purpura; *IVIG* intravenous immunoglobulin; *LPD* lymphoproliferative disease; *M* male; *MMF* mycophenolate mofetil; *MTX* methotrexate; *ND* not described; *PID* primary immnodeficiency disease; *RA* rheumatoid arthritis, *Ref* reference; *Tac* tacrolimus; *UC* ulcerative colitis

It has been reported that most patients with EBV-MCU had a benign course after the dose reduction or discontinuation of immunosuppressive agents or during the follow-up [2, 4, 5, 9, 10, 13]. According to the summary of 51 patients with EBV-MCU reported by Roberts et al., 19 of the 45 patients with complete treatment records were treated only by reducing the doses of immunosuppressive agents, and 17 of them attained a complete remission. Moreover, 11 patients received no therapeutic intervention and six of them showed spontaneous regression [9]. However, the factors that contributed to the decision of a therapeutic strategy and the prognosis prediction were not clarified. All 14 patients, including our patient, with gastrointestinal tract EBV-MCU received some form of treatment. Four patients, including our patient, underwent resection of the lesion site [3, 11, 12]. The reasons for the resection were perforation at the lesion site in one patient, the absence of improvement of clinical symptoms or conditions after the reduction of doses of immunosuppressive agents or during the natural course of the disease in two patients, and intestinal obstruction in our patient [3, 11, 12]. The intestinal obstruction in our patient probably developed as a result of repeated remission and exacerbation of the lesion site during the natural course of the disease, which caused persistent inflammation over a long period, leading to marked fibrosis. This is the first and very valuable report on a patient with gastrointestinal tract EBV-MCU that developed into an intestinal obstruction. On the basis of previous reports and this report, gastrointestinal tract EBV-MCU may develop into a tumorous lesion or an ulcerative lesion or even intestinal obstruction. Active therapeutic intervention may be required for such a lesion.

It is important to distinguish EBV-MCU from malignant lymphomas, especially Hodgkin lymphoma and DLBCL. In the present case, EBV-MCU was distinguished from a malignant lymphoma on the basis of histopathological and immunohistological findings of the specimen resected from the lesion site as well as flow cytometry and G-banding differential staining findings. Because the therapeutic strategy for EBV-MCU may largely differ from those for malignant lymphomas, it is necessary to know EBV-MCU as a new disease concept and carefully examine tumorous lesions in the gastrointestinal tract taking EBV-MCU into consideration.

Although the long-term prognosis of gastrointestinal tract EBV-MCU still remains largely unclarified, it has been reported that most patients had a benign long-term course [2, 4, 5, 11, 12]. At present, there has been no proposal on the recommended intervals and duration of catamnestic follow-up. On the other hand, it has been reported that a patient with EBV-MCU who had received immunosuppressive agents for Crohn’s disease developed Hodgkin lymphoma 18 months after discontinuation of immunosuppressive agents [7]. Regarding the relationship between lymphoma and inflammatory bowel disease (IBD) including Crohn’s disease, it is assumed that the risk of developing lymphoma is increased by immunosuppressive therapy in IBD, especially in patients treated with thiopurines and thiopurines in combination with tumor necrotic factor (TNF)-α [15, 16]. IBD patients treated with immunosuppressive agents are considered to be at a high risk of developing EBV-MCU [2–13]. Furthermore, it is suspected that EBV-MCU may be an early polyclonal EBV-driven lymphoproliferative disease that may progress to lymphoma, according to previous reports on immunoglobulin heavy-chain (IgH) gene rearrangement [7, 17]. Thus, it is suspected that EBV-MCU may be involved in some cases of lymphomas arising from IBD. Careful observation is recommended even after the improvement of clinical conditions, considering not only the possibility of recurrence but also the risk of transformation to a malignant lymphoma.

EBV-MCU is a new disease concept [1] and is far from being widely recognized. In the past, there may have been cases in which an ulcerative lesion occurring in the gastrointestinal tract was EBV-MCU but was not correctly diagnosed because of the low recognition of EBV-MCU. In an aging society, it is suspected that the number of patients with EBV-MCU will increase with an increased incidence. Further accumulation of cases may clarify the clinical conditions of EBV-MCU.

We experienced treating an elderly male patient with gastrointestinal tract EBV-MCU that developed into an intestinal obstruction and required surgical resection. The number of patients with EBV-MCU will increase with the aging of society and increasing use of immunosuppressive therapy. It is necessary to consider the possibility of EBV-MCU when examining an ulcerative or tumorous lesion in the gastrointestinal tract.

## Data Availability

All the data supporting our findings is contained within the manuscript.

## Abbreviations

*Adm*: Administration
*AZA*: Azathioprine
*CA19–9*: carcinoantigen 19–9
*CD*: Crohn’s disease
*CEA*: Carcinoembryonic antigen
*CKD*: Chronic kidney disease
*CT*: Computed tomography
*CyA*: Cyclosporine
*DLBCL*: diffuse large B-cell lymphoma
*EBER-1*: Epstein–Barr virus-encoded small RNA-1
*EBV-MCU*: Epstein Barr virus-positive mucocutaneous ulcer
*F*: Female
*HL*: Hodgkin’s lymphoma
*HRS*: Hodgkin and Reed–Sternberg
*HSCT*: Hematopoietic stem cell transplantation
*IgA*: Immunoglobulin A
*IgG*: Immunoglobulin G
*IgM*: Immunoglobulin M
*IL-2R*: Interleukin-2 receptor
*ISH*: In situ hybridization
*ITP*: Idiopathic thrombocytopenic purpura
*IVIG*: Intravenous immunoglobulin
*LPD*: Lymphoproliferative disease
*M*: Male
*MMF*: Mycophenolate mofetil
*MTX*: Methotrexate
*ND*: Not described
*PID*: Primary immnodeficiency disease
*RA*: Rheumatoid arthritis
*Ref*: Reference
*Tac*: Tacrolimus
*TCS*: Total colonoscopy
*UC*: Ulcerative colitis

## References

[1] Swerdlow SH, Campo E, Pileri SA, Harris NL, Stein H, Siebert R, Advani R, Ghielmini M, Salles GA, Zelenetz AD, Jaffe ES (2016). The 2016 revision of the World Health Organization classification of lymphoid neoplasms. Blood.

[2] Dojcinov SD, Venkataraman G, Raffeld M, Pittaluga S, Jaffe ES (2010). EBV positive mucocutaneous ulcer – a study of 26 cases associated with various sources of immunosuppression. Am J Surg Pathol.

[3] Di Napoli A, Giubettini M, Duranti E, Ferrari A, Guglielmi C, Uccini S, Ruco L (2011). Iatrogenic EBV-positive lymphoproliferative disorder with features of EBV+ mucocutaneous ulcer: evidence for concomitant TCRγ/IGH rearrangements in the Hodgkin-like neoplastic cells. Virchows Arch.

[4] Hart M, Thakral B, Yohe S, Balfour HH, Singh C, Spears M, McKenna RW (2014). EBV-positive mucocutaneous ulcer in organ transplant recipients: a localized indolent posttransplant lymphoproliferative disorder. Am J Surg Pathol.

[5] Matnani R, Peker D (2014). Azathioprine induced Epstein Barr virus-positive mucocutaneous ulcer arising in perianal fistula and abscess associated with Crohn's disease. J Crohns Colitis.

[6] Kleinman S, Jhaveri D, Caimi P, Cameron R, Lemonovich T, Meyerson H, Hostoffer R, Tcheurekdjian H (2014). A rare presentation of EBV^+^ mucocutaneous ulcer that led to a diagnosis of hypogammaglobulinemia. J Allergy Clin Immunol Pract.

[7] Moran NR, Webster B, Lee KM, Trotman J, Kwan YL, Napoli J, Leong RW (2015). Epstein Barr virus-positive mucocutaneous ulcer of the colon associated Hodgkin lymphoma in Crohn's disease. World J Gastroenterol.

[8] Bunn B, van Heerden W (2015). EBV-positive mucocutaneous ulcer of the oral cavity associated with HIV/AIDS. Oral Surg Oral Med Oral Pathol Oral Radiol.

[9] Roberts TK, Chen X, Liao JJ (2016). Diagnostic and therapeutic challenges of EBV-positive mucocutaneous ulcer: a case of report and systematic review of the literature. Exp Hematol Oncol.

[10] Satou A, Kohno A, Fukuyama R, Elsayed AA, Nakamura S (2017). Epstein-Barr virus-positive mucocutaneous ulcer arising in a post-hematopoietic cell transplant patient followed by polymorphic posttransplant lymphoproliferative disorder and cytomegalovirus colitis. Hum Pathol.

[11] Osman M, Al Salihi M, Abu Sitta E, Al Hadidi S. A rare case of Epstein-Barr virus mucocutaneous ulcer of the colon. BMJ Case Rep. 2017 Jul 6;2017. pii: bcr-2017-bc220717. doi: 10.1136/bcr-2017-220717. 10.1136/bcr-2017-220717 PMC5535207 28687701

[12] Juan A, Lobatón T, Tapia G, Mañosa M, Cabré E, Domènech E (2017). Epstein-Barr virus-positive mucocutaneous ulcer in Crohn's disease. A condition to consider in immunosuppressed IBD patients. Dig Liver Dis.

[13] Hujoel IA, Rubio-Tapia A, Dao LN, Porrata LF, Kane SV (2018). Epstein-Barr virus-positive Mucocutaneous ulcer in an immunosuppressed patient. ACG Case Rep J.

[14] Betjes MG (2013). Immune cell dysfunction and inflammation in end-stage renal disease. Nat Rev Nephrol.

[15] Sokol H, Beaugerie L, Maynadié M, Laharie D, Dupas JL, Flourié B, Lerebours E, Peyrin-Biroulet L, Allez M, Simon T, Carrat F, Brousse N, CESAME Study Group (2012). Excess primary intestinal lymphoproliferative disorders in patients with inflammatory bowel disease. Inflamm Bowel Dis.

[16] Kotlyar DS, Lewis JD, Beaugerie L, Tierney A, Brensinger CM, Gisbert JP, Loftus EV, Peyrin-Biroulet L, Blonski WC, Van Domselaar M, Chaparro M, Sandilya S, Bewtra M, Beigel F, Biancone L, Lichtenstein GR (2015). Risk of lymphoma in patients with inflammatory bowel disease treated with azathioprine and 6-mercaptopurine: a meta-analysis. Clin Gastroenterol Hepatol.

[17] Kumar S, Fend F, Quintanilla-Martinez L, Kingma DW, Sorbara L, Raffeld M, Banks PM, Jaffe ES (2000). Epstein-Barr virus-positive primary gastrointestinal Hodgkin's disease: association with inflammatory bowel disease and immunosuppression. Am J Surg Pathol.

